# Supplementary material for: Comparative genomics provides new insights into the diversity, physiology, and sexuality of the only industrially exploited tremellomycete: Phaffia rhodozyma
Source: BMC Genomics. 2016 Nov 9;17:901. doi: 10.1186/s12864-016-3244-7 (PMC5103461; doi:10.1186/s12864-016-3244-7)
Supplement: Additional file 6: — List of orphan genes with links to PFAM (related to Additional file 1: Table S1). (ZIP 1428 kb) [file 12864_2016_3244_MOESM6_ESM.zip › BLAST_HTML_FTR/G03036_P.html]

BLAST Search Results


```
BLASTP 2.2.27+


Reference:
Stephen F. Altschul, Thomas L. Madden, Alejandro A. Schäffer,
Jinghui Zhang, Zheng Zhang, Webb Miller, and David J. Lipman (1997),
"Gapped BLAST and PSI-BLAST: a new generation of protein database
search programs", Nucleic Acids Res. 25:3389-3402.


Reference for
composition-based statistics:
Alejandro A. Schäffer, L. Aravind, Thomas L. Madden, Sergei
Shavirin, John L. Spouge, Yuri I. Wolf, Eugene V. Koonin, and
Stephen F. Altschul (2001), "Improving the accuracy of PSI-BLAST
protein database searches with composition-based statistics and
other refinements", Nucleic Acids Res. 29:2994-3005.


Database: nr
           71,551,133 sequences; 26,053,659,533 total letters


Query= G03036_P

Length=757
                                                                      Score     E
Sequences producing significant alignments:                          (Bits)  Value

emb|CED84405.1|  hypothetical protein [Xanthophyllomyces dendrorh...  1476    0.0  


 >emb|CED84405.1| hypothetical protein [Xanthophyllomyces dendrorhous]
Length=756

 Score = 1476 bits (3822),  Expect = 0.0, Method: Compositional matrix adjust.
 Identities = 753/756 (99%), Positives = 754/756 (99%), Gaps = 0/756 (0%)

Query  1    MPSSSLRPSTKINKSQTPARTKEKSTTEPDQKRISVHRRSSSGVTSKKSVDQMTTSSPHS  60
            MPSSSLRPSTKINKSQTPARTKEKSTTEPDQKRISVHRRSSSGVTSKKSVDQMTTSSPHS
Sbjct  1    MPSSSLRPSTKINKSQTPARTKEKSTTEPDQKRISVHRRSSSGVTSKKSVDQMTTSSPHS  60

Query  61   THKDNPSLAKGVVGKSLAPISSRVRRMTTRSSIKSLQSQPSDSTSLSSPSANQQDTHAEA  120
            THKDNPSLAKGVVGKSLAPISSRVRRMTTRSSIKSLQSQPSDSTSLSSPSANQQDTHAEA
Sbjct  61   THKDNPSLAKGVVGKSLAPISSRVRRMTTRSSIKSLQSQPSDSTSLSSPSANQQDTHAEA  120

Query  121  SPTLISEEESATMALIASTIPKASTNLPSRKSNIPISRPASTASLFANPSSRAPAFAHRR  180
            SPTLISEEESATMALIASTIPKAST LPSRKSNIPISRPASTASLFANPSSRAPAFAHRR
Sbjct  121  SPTLISEEESATMALIASTIPKASTTLPSRKSNIPISRPASTASLFANPSSRAPAFAHRR  180

Query  181  SISLAFASPPSSALEQSDDSSPPSLGPPMTTTPRSLRRSVSSSFDSPSNLVISTPSDPTP  240
            SISLAFASPPSSALEQSDDSSPPSLGPPMTTTPRSLRRSVSSSFDSPSNLVISTPSDPTP
Sbjct  181  SISLAFASPPSSALEQSDDSSPPSLGPPMTTTPRSLRRSVSSSFDSPSNLVISTPSDPTP  240

Query  241  DISSSPSRVTAIPKISMTPSPIKISRPVVRSLVPKTSSSSSGRPSTVSGSLAFLAVQNDS  300
            DISSSPSRVTAIPKISMTPSPIKISRPVVRSLVPKTSSSSSGRPSTVSGSLAFLAVQNDS
Sbjct  241  DISSSPSRVTAIPKISMTPSPIKISRPVVRSLVPKTSSSSSGRPSTVSGSLAFLAVQNDS  300

Query  301  SRSTPRNQSIPILKKGHRPSLSTSTVPSKSANSLGRKRPDSVSITVTSTSLNNKSANTPH  360
            SRSTPRNQSIPILKKGHRPSLSTSTVPSKSANSLGRKRPDSVSITVTSTSLNNKSANTPH
Sbjct  301  SRSTPRNQSIPILKKGHRPSLSTSTVPSKSANSLGRKRPDSVSITVTSTSLNNKSANTPH  360

Query  361  SVSSPGKLSGRSEKADLKRTPIKEVNSEDSMDEISVSSPVVEKVSVVEKTRRVIPKSPLK  420
            SVSSPGKLSGRSEKADLKRTPIKEVNSEDSMDEISVSSPVVEKVSVVEKTRRVIPKSPLK
Sbjct  361  SVSSPGKLSGRSEKADLKRTPIKEVNSEDSMDEISVSSPVVEKVSVVEKTRRVIPKSPLK  420

Query  421  EMTEDGVDCSPLHHTDRPFPSLYPPNQSSSSQMLKNLLTPSATPYGSRVRPRPLVARNSL  480
            EMTEDGVDCSPLHHTDRPFPSLYPPNQSSSSQMLKNLLTPSATPYGSRVRPRPLVARNSL
Sbjct  421  EMTEDGVDCSPLHHTDRPFPSLYPPNQSSSSQMLKNLLTPSATPYGSRVRPRPLVARNSL  480

Query  481  VHFDSMAQSFALEDEGDSLLHLPEDGPNLSFPDLSGPDLESPPGSLKRKARMRPSLVAPT  540
            VHFDSMAQSFALEDEGDSLLHLPEDGPNLSFPDLSGPDLESPPGSLKRKARMRPSLVAPT
Sbjct  481  VHFDSMAQSFALEDEGDSLLHLPEDGPNLSFPDLSGPDLESPPGSLKRKARMRPSLVAPT  540

Query  541  KAKEHAKLVSLQDRLDESLLAQTALSELLEREIGKVRELEEHLAERQIEEDLLKASPVKP  600
            KAKEHAKLVSLQDRLDESLLAQTALSELLEREIGKVRELEEHLAERQIEEDLLKASPVKP
Sbjct  541  KAKEHAKLVSLQDRLDESLLAQTALSELLEREIGKVRELEEHLAERQIEEDLLKASPVKP  600

Query  601  SAPTFSRPFLGKPMTEVEAVVVQENDLRLKTEVARLKIELESSTDCIEHLQTGLSELTEQ  660
            SAPTFSRPFLGKPMTEVEAVVVQENDLRLKTEV RLKIELESSTDCIEHLQTGLSELTEQ
Sbjct  601  SAPTFSRPFLGKPMTEVEAVVVQENDLRLKTEVTRLKIELESSTDCIEHLQTGLSELTEQ  660

Query  661  LTSAHGDIDSLQHDLISIQSKNSRLEADNVSREAELEAQQIASTVEGRKKASKAWSEVKM  720
            LTSAHGDIDSLQHDLISIQSKNS+LEADNVSREAELEAQQIASTVEGRKKASKAWSEVKM
Sbjct  661  LTSAHGDIDSLQHDLISIQSKNSKLEADNVSREAELEAQQIASTVEGRKKASKAWSEVKM  720

Query  721  RAMGELEDLKGERDLLAILETSIRLLEREVRSGRTI  756
            RAMGELEDLKGERDLLAILETSIRLLEREVRSGRTI
Sbjct  721  RAMGELEDLKGERDLLAILETSIRLLEREVRSGRTI  756


Lambda      K        H        a         alpha
   0.307    0.122    0.326    0.792     4.96 

Gapped
Lambda      K        H        a         alpha    sigma
   0.267   0.0410    0.140     1.90     42.6     43.6 

Effective search space used: 8776863572828


  Database: nr
    Posted date:  Sep 23, 2015 12:05 AM
  Number of letters in database: 26,053,659,533
  Number of sequences in database:  71,551,133


Matrix: BLOSUM62
Gap Penalties: Existence: 11, Extension: 1
Neighboring words threshold: 11
Window for multiple hits: 40
```
